# Hypocretin/orexin regulation of dopamine signaling: implications for reward and reinforcement mechanisms

**DOI:** 10.3389/fnbeh.2012.00054

**Published:** 2012-08-21

**Authors:** Erin S. Calipari, Rodrigo A. España

**Affiliations:** ^1^Department of Physiology and Pharmacology, Wake Forest School of MedicineWinston Salem, NC, USA; ^2^Department of Neurobiology and Anatomy, Drexel University College of MedicinePhiladelphia, PA, USA

**Keywords:** hypocretin, orexin, cocaine, voltammetry, reward, self-administration, ventral tegmental area

## Abstract

The hypocretins/orexins are comprised of two neuroexcitatory peptides that are synthesized exclusively within a circumscribed region of the lateral hypothalamus. These peptides project widely throughout the brain and interact with a variety of regions involved in the regulation of arousal-related processes including those associated with motivated behavior. The current review focuses on emerging evidence indicating that the hypocretins influence reward and reinforcement processing via actions on the mesolimbic dopamine system. We discuss contemporary perspectives of hypocretin regulation of mesolimbic dopamine signaling in both drug free and drug states, as well as hypocretin regulation of behavioral responses to drugs of abuse, particularly as it relates to cocaine.

## Brief introduction to the hypocretin/orexin system

The hypocretin/orexin system consists of two neuroexcitatory peptides (hypocretin-1 and hypocretin-2) that are synthesized within neurons restricted to the lateral hypothalamus and adjacent regions. These neurons project to a vast number of brain regions and interact with two known G-protein-coupled receptors, the hypocretin 1 and hypocretin 2 receptors (de Lecea et al., 1998; Sakurai et al., 1998; Zhu et al., 2003). Both hypocretin receptors are expressed widely throughout the brain although their expression levels vary based on location (Trivedi et al., 1998; Bourgin et al., 2000; Greco and Shiromani, 2001; Hervieu et al., 2001; Marcus et al., 2001; Backberg et al., 2002; Cluderay et al., 2002; Suzuki et al., 2002). The widespread distribution patterns of fibers and receptors positions the hypocretin system to interact with a variety of neural structures known to be involved in the regulation of arousal-related processes. It is posited that the hypocretins modulate a number of cognitive, affective, and homeostatic processes associated with arousal via these interactions (Peyron et al., 1998; Piper et al., 2000; España et al., 2001; Adamantidis et al., 2007).

Over the past decade, a series of studies has expanded our understanding of hypocretin involvement in arousal-related processes and their influence on motivated behavior, reward/reinforcement, and the neural mechanisms underlying these actions. These studies have demonstrated that enhancement of hypocretin signaling promotes neurochemical and behavioral responses to drugs of abuse, while manipulations that reduce hypocretin signaling disrupt these responses. The present review will focus on hypocretin regulation of baseline and cocaine-induced changes in dopamine signaling as well as motivated behaviors reliant on dopamine neurotransmission.

## Hypocretin regulates baseline levels of dopamine signaling

Accumulating evidence indicates that the hypocretin system regulates dopamine function via direct actions on hypocretin receptors within the ventral tegmental area (VTA) where a large population of dopamine neurons resides. The first evidence for this came from anatomical studies showing a significant hypocretin innervation of the VTA where both hypocretin 1 and hypocretin 2 receptors are found on dopamine neurons (Marcus et al., 2001; Fadel and Deutch, 2002; Baldo et al., 2003; Narita et al., 2006). Consistent with these observations, hypocretins increase tonic and burst firing of dopamine neurons in the VTA, further signifying that hypocretins regulate dopamine function (Korotkova et al., 2003). Conversely, blockade of hypocretin 1 receptors reduces dopamine cell firing (Moorman and Aston-Jones, 2010).

Although there is substantial evidence for direct excitatory actions of hypocretins throughout the brain, including on dopamine neurons of the VTA (Ivanov and Aston-Jones, 2000; Korotkova et al., 2003, 2006; Moorman and Aston-Jones, 2010), several observations suggest that hypocretins also facilitate glutamatergic excitation of VTA dopamine neurons. For instance, Borgland and colleagues have shown elegantly that both hypocretin-1 and -2 augment glutamatergic excitability of dopamine neurons (Borgland et al., 2006, 2008), likely via hypocretin-induced increases in synaptic NMDA receptors within the VTA (Borgland et al., 2006). Importantly, blockade of hypocretin 1 receptors using SB-334867 reduces the hypocretin-1-mediated enhancement of NMDA receptor currents of VTA dopamine neurons (Borgland et al., 2006). Other evidence for hypocretin facilitation of glutamate-mediated excitation of VTA dopamine neurons comes from experiments where VTA neuronal firing was elicited by stimulation of the medial prefrontal cortex which provides significant glutamatergic innervation to the VTA. When delivered directly into the VTA, hypocretin-1 potentiated dopamine cell firing elicited by cortex stimulation, indicating that synaptic connections between cortical glutamatergic neurons and dopamine cell bodies are responsive to hypocretins (Moorman and Aston-Jones, 2010). When taken together, these observations provide strong evidence for hypocretin regulation of dopaminergic firing, in part, by facilitating glutamatergic signaling within the VTA.

The consequences of hypocretin-mediated alterations in dopamine neuronal activity are evident in a series of studies investigating the effects of hypocretins on synaptic dopamine transmission under baseline conditions. Initial studies using microdialysis have demonstrated inconsistent effects of hypocretin on tonic dopamine levels. For instance, one study using hypocretin-1 infusions into the VTA showed marked increases in dopamine levels in the NAc, although it is unclear to what extent the NAc core or shell was targeted (Narita et al., 2006). In another study, hypocretin-1 infusions into the VTA failed to show increases in dopamine within the NAc core, but did show dopamine elevations in the prefrontal cortex and the NAc shell (Vittoz and Berridge, 2006; Vittoz et al., 2008). Consistent with this latter observation, in recent studies we have also shown a lack of hypocretin-1 effect on baseline dopamine signaling in the NAc core (España et al., 2011).

Despite the inconsistencies observed with experiments testing the effects of enhanced hypocretin neurotransmission on dopamine signaling, it remained possible that some degree of hypocretin tone was necessary for normal dopamine function. To examine this, we again used microdialysis to test the effects of hypocretin 1 receptor blockade on dopamine signaling in the NAc core (España et al., 2010). These studies used the selective hypocretin 1 receptor antagonists, SB-334867 which has 50-fold selectivity for the hypocretin 1 receptor over the hypocretin 2 receptor (Smart et al., 2001). Additionally, this antagonist has been reported to have no appreciable selectivity for over 50 other G-protein coupled receptors and ion channels (Smart et al., 2001). On testing days, rats were pretreated with i.p. vehicle or 30 mg/kg SB-334867 and dopamine levels were sampled for thirty minutes before the animal received a second manipulation (see below for description of cocaine studies). As might be predicted from the lack of effects observed with previous hypocretin-1 studies, i.p. injections of SB-334867 had little effect on dopamine levels under baseline conditions, although a small trend for reduced dopamine was observed. These observations are in agreement with another recent microdialysis study in which subcutaneous SB-334867 injections did not alter baseline levels of dopamine in the NAc shell (Quarta et al., 2010). Interestingly, however, we repeated these studies with the exception that rats were treated with SB-334867 (10 nmol) directly into the VTA. Using this approach, blockade of hypocretin 1 receptors significantly reduced dopamine levels in the NAc core (España et al., 2010). Although it is unclear why i.p. injections of SB-334867 failed to reduce dopamine signaling, the fact that intra-VTA SB-334867 infusions significantly reduced baseline dopamine suggests that hypocretin neurotransmission within this region is important for normal dopamine signaling.

**Figure 1:**
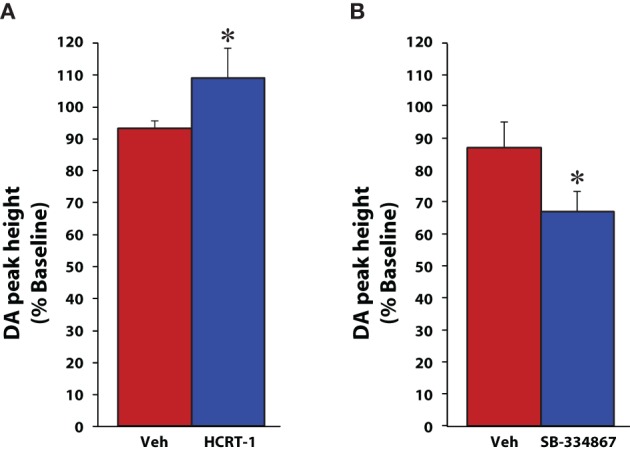
**Hypocretin manipulations influence evoked dopamine release under baseline conditions.** Shown are the mean ± SEM of peak heights of stimulated dopamine (DA) release for rats that received **(A)** vehicle (*n* = 6) or 1 nmol hypocretin-1 (*n* = 6) into the VTA, or **(B)** vehicle (*n* = 8) or 10 nmol SB-334867 (*n* = 6) into the VTA. Compared to vehicle, hypocretin-1 increased, while SB-334867 reduced the peak height of stimulated dopamine release in the NAc core. ^*^ *P* < 0.05 relative to vehicle. Modified from España et al. (2010, 2011).

Although microdialysis is a useful technique, it suffers from relatively low temporal resolution and solely provides information on tonic changes in dopamine signaling that typically occur over extended periods of time (typically 10–20 min sampling). In contrast, the high temporal resolution afforded by fast scan cyclic voltammetry allows for rapid sampling of phasic changes in both dopamine release and uptake. Using voltammetry in anesthetized rats we further explored the possibility that hypocretin regulates dopamine signaling (España et al., 2010; 2011). Rats were implanted with a recording electrode in the NAc core and an infusion cannula affixed to a stimulating electrode in the VTA. Under these conditions, electrical stimulation of the VTA elicits consistent action-potential-mediated dopamine efflux which can be used to measure changes in the amplitude of dopamine release (peak height in μM) as well as the rate of dopamine uptake (*V*_max_) via the dopamine transporter. In initial studies, rats were pretreated with vehicle or hypocretin-1 (0.5 nmol) directly into the VTA and dopamine signaling was recorded for 20 min prior to additional manipulations (see cocaine results below). In contrast to what was observed with the microdialysis studies, hypocretin-1 significantly increased evoked-dopamine release within 5 min of administration without affecting dopamine uptake rate (Figure 1A; España et al., 2011).

Consistent with these findings, disruption of hypocretin neurotransmission produces the opposite effects. Thus, in another set of studies, rats received an infusion of vehicle or SB-334867 (10 nmol) directly into the VTA and dopamine responses were monitored for 40 mins (España et al., 2010). Relative to vehicle-treated rats, SB-334867 produced a significant reduction in stimulated dopamine release, further indicating that hypocretin signaling regulates dopamine neurotransmission (Figure 1B). Additionally, in a final set of experiments, we used *in vitro* voltammetry in brain slices from wild type (WT) and hypocretin knockout (KO) mice to examine whether a complete loss of hypocretin signaling would produce deficits in dopamine signaling. Hypocretin KO and WT mice were sacrificed and recording and stimulating electrodes were positioned within the NAc core to allow for measurement of locally-evoked dopamine release (España et al., 2010). Table 1 shows that under baseline conditions, hypocretin KO mice displayed reductions in both evoked dopamine release and dopamine uptake rate, again indicating that hypocretin neurotransmission is necessary to maintain normal levels of dopamine signaling.

**Table 1:** **Hypocretin KO mice show disrupted dopamine signaling under baseline conditions and in response to cocaine**.

**Group**	**Baseline [DA_p_]**	**Baseline *V*_max_**	**Uptake Inhibition (apparent affinity K_m_)**
WT	1.69 ± 2.5 μM	3.5 ± 0.4 μM/s	22.5 ± 3.5 μM
KO	1.04 ± 2.2 μM^*^	2.1 ± 0.3 μM/s^**^	10.9 ± 1.2 μM^**^

Shown are mean ± SEM for baseline levels of dopamine release [DA_p_] and maximal uptake rate (V_max_), as well as cocaine-induced dopamine uptake inhibition (apparent affinity K_m_) for WT and hypocretin KO mouse slices containing the NAc core. Under baseline conditions, hypocretin KO mice show reduced dopamine release and reduced dopamine uptake rates relative to WT mice. Following superfusion of 30 uM cocaine, cocaine-induced dopamine uptake inhibition was significantly lower in hypocretin KO mice.

* P < 0.05;

** P < 0.01. Modified from España et al. (2010).

### Summary

Despite somewhat conflicting observations, the neurochemical studies described above indicate that the hypocretin system influences some aspects of dopamine signaling under normal, baseline conditions. Microdialysis studies suggest that hypocretin signaling regulates dopamine levels in the prefrontal cortex and possibly the NAc shell, while voltammetry studies show that hypocretin signaling is necessary to maintain normal levels of dopamine release within the NAc core. Together, these observations offer further support for the hypothesis that the hypocretin system participates in the regulation of dopamine signaling and that hypocretin actions on dopamine systems could influence behaviors known to be regulated by dopamine.

## Hypocretin regulates drug-induced changes in dopamine signaling

In addition to regulating dopamine signaling under baseline conditions, hypocretins also exert a profound influence on dopamine responses to drugs of abuse. For example, in brain slices containing the VTA, hypocretin-1 enhanced cocaine-induced potentiation of glutamatergic currents (AMAPA/NMDAR ratio) in dopamine neurons in animals that had received non-contingent cocaine injections. Importantly, these effects were blocked with SB-334867 (Borgland et al., 2006). Moreover, a recent study demonstrates that a history of cocaine self-administration preferentially enhances hypocretin-1 potentiation of NMDA receptor plasticity in VTA neurons (Borgland et al., 2009). The hypocretin-mediated augmentation in VTA gultamatergic transmission appears to occur selectively with highly salient reinforcers such as cocaine and high fat food pellets as this potentiation did not occur following aversive stimuli such as foot shock (Borgland et al., 2009).

**Figure 2:**
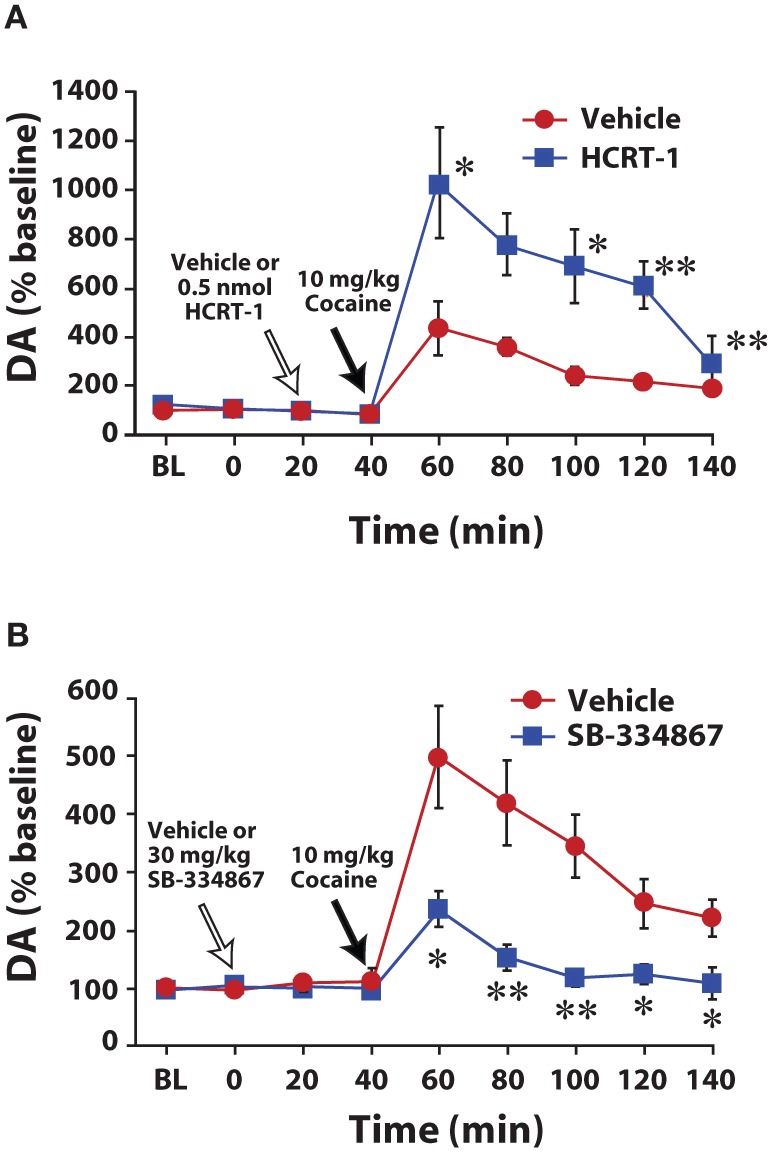
**Hypocretin manipulations influence cocaine-induced elevations in extracellular dopamine within the NAc core. (A)** Shown are the mean ± SEM of extracellular levels of dopamine (DA) within the NAc core following intra-VTA infusion of vehicle (*n* = 6) or 0.5 nmol hypocretin-1 (HCRT-1; *n* = 6). **(B)** Shown are the mean ± SEM of extracellular levels of dopamine (DA) within the NAc core following i.p. injection of vehicle (*n* = 6) or 30 mg/kg SB-334867 (*n* = 6). ^*^P < 0.05, ^**^P < 0.01 relative to vehicle. Modified from España et al. (2010, 2011).

In a series of neurochemical studies we examined the effects of hypocretin manipulations on dopamine responses to cocaine. In initial experiments, rats were implanted for microdialysis sampling in the NAc core and dopamine levels were measured in response to treatment with hypocretin agents and cocaine. Rats were pretreated with vehicle or hypocretin-1 (0.5 nmol) directly into the VTA 20 min prior to receiving a single 10 mg/kg injection of cocaine. In rats treated with vehicle, injections of cocaine produced expected increases in extracellular dopamine levels. In contrast, hypocretin-1 significantly augmented the effects of cocaine to nearly double of what was observed with vehicle (Figure 2A; España et al., 2011). The opposite effect was observed when hypocretin neurotransmission was compromised. Thus, rats pretreated with i.p. or intra-VTA SB-334867 showed significantly lower levels of dopamine in response to cocaine (Figure 2B; España et al., 2010). These data are consistent with another set of studies showing that i.p. SB-334867 reduces the effects of amphetamine on dopamine levels in the NAc shell (Quarta et al., 2010) and that hypocretin KO mice show reduced extracellular dopamine responses to morphine and cocaine (Narita et al., 2006; España and Jones^2010^).

**Figure 3:**
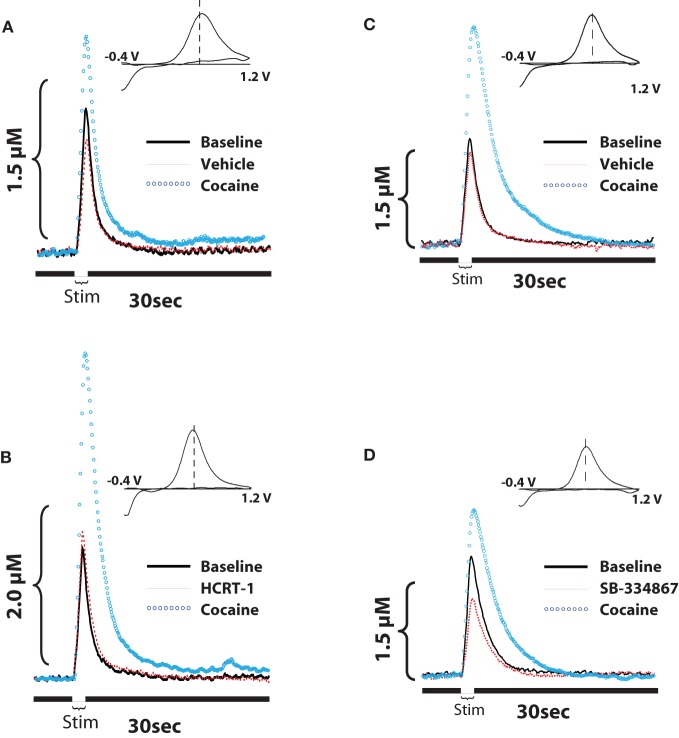
**Hypocretin signaling influences cocaine-induced changes in evoked-dopamine release and uptake in the NAc core. (A,B)** Shown are representative concentration-time plots and cyclic voltammograms (insets) of dopamine responses from rats that received pretreatment infusions of vehicle or 0.5 nmol hypocretin-1 into the VTA. **(C,D)** Shown are representative concentration-time plots and cyclic voltammograms (insets) of dopamine responses from rats that received pretreatment infusions of vehicle or 10 nmol SB-334867 into the VTA. Stim represents the time of electrical stimulation (1 s, 60 Hz pulse). (Insets in **A–D**) Cyclic voltammograms depict two current peaks, one at 600 mV (positive deflection) for dopamine oxidation and one at –200 mV (negative deflection) for reduction of dopamine-o-quinone. The position of the peaks identifies the substance oxidized as dopamine. Compared to their respective vehicle groups, hypocretin-1 augments, while SB334867 reduces, the effects of cocaine on both evoked dopamine release and dopamine uptake inhibition. Modified from España et al. (2010, 2011).

A similar set of results was also obtained in studies using voltammetry in anesthetized rats (España et al., 2010, 2011). As described above, rats were implanted with a recording electrode in the NAc core, an infusion cannula/stimulating electrode in the ipsilateral VTA, and an i.v. jugular catheter for delivery of cocaine. On testing days, rats were pretreated with vehicle or hypocretin-1 (0.5 nmol) into the VTA 20 min prior to receiving 1.5 mg/kg i.v. cocaine. As shown in Figures 3A,B, within 30 s of cocaine delivery, hypocretin-1 pretreated animals showed significantly greater evoked dopamine release than animals treated with vehicle (España et al., 2011). Moreover, hypocretin-1 also augmented cocaine-induced uptake inhibition suggesting that dopamine neurons were more sensitive to the effects of cocaine. As expected, voltammetry experiments using SB-334867 showed the opposite effects (España et al., 2010). Rats received an infusion of vehicle or SB-334867 (10 nmol) directly into the VTA 40 minutes prior to i.v. delivery of cocaine (1.5 mg/kg). When compared to vehicle-treated animals, SB-334867 significantly reduced the effects of cocaine on both dopamine peak height and dopamine uptake inhibition, signifying that cocaine was no longer as effective at inhibiting dopamine uptake (Figures 3C,D). Consistent with these observations, *in vitro* voltammetry in brain slices from WT and hypocretin KO mice demonstrate that in addition to baseline disruptions in dopamine signaling, a loss of hypocretin also reduces the effects of cocaine on evoked dopamine release and dopamine uptake inhibition (España et al., 2010).

## Potential mechanisms underlying hypocretin modulation of dopamine signaling

Although the mechanisms involved in hypocretin regulation of dopamine neurotransmission are not yet understood, there is evidence that hypocretins may influence baseline dopamine signaling and dopamine responses to drugs of abuse by altering the activity state of dopamine neurons in the VTA. As mentioned above, hypocretins promote glutamatergic enhancement of excitatory synaptic transmission in dopamine neurons of the VTA (Borgland et al., 2006, 2009), and in our voltammetry studies we show that hypocretin agents within the VTA alter dopamine signaling under baseline conditions and following cocaine (España et al., 2010, 2011). These effects are consistent with previous work indicating that hypocretin manipulations alter dopamine cell firing (Ivanov and Aston-Jones, 2000; Korotkova et al., 2003, 2006; Moorman and Aston-Jones, 2010). We posit that by altering the activity state of dopamine neurons, hypocretins not only affect dopamine transmission directly, but may also exert effects that serve to alter the sensitivity of dopamine systems to drug treatments. In terms of cocaine, observed increases in dopamine levels within terminal regions are known to be associated with blockade of the dopamine transporter (Ritz et al., 1987). Nevertheless, recent observations indicate that in addition to blocking dopamine uptake, cocaine stimulates glutamate release in the VTA (Wise et al., 2008) and increases the incidence and/or magnitude of dopamine release events in the NAc shell (Aragona et al., 2008). These two latter observations provide additional mechanisms for increased dopamine signaling that may not be directly reliant on dopamine transporter function. Based on this evidence, it is possible that by altering baseline dopamine activity via changes in dopamine neuron firing or sensitivity to glutamate, hypocretin manipulations could induce dopamine neurons to display a differential sensitivity to cocaine and possibly other drugs of abuse.

Another possibility is that the hypocretin system influences dopamine signaling by altering the functional state of dopamine terminals. The dopamine transporter can be modulated via a number of second messenger signaling cascades that result in phosphorylation and glycosylation, both of which can alter the stability of the dopamine transporter in the membrane (Li et al., 2004; Johnson et al., 2005; Mortensen et al., 2008). Given that baseline uptake rates are dependent on functional dopamine transporters, modifications that alter dopamine transporter levels at the cell surface result in changes to baseline dopamine uptake rates, an effect that can alter psychostimulant potency. In addition, dopamine transporter trafficking to the membrane can occur rapidly, in as little as 10 s, therefore it is not surprising that acute treatment with hypocretin agents can alter the potency of psychostimulants such as cocaine (Furman et al., 2009). One explanation for how hypocretin manipulations in the VTA can alter dopamine signaling in dopamine terminal regions is through hypocretin's ability to alter dopamine release. By altering dopamine levels at the synapse, hypocretin manipulations can influence activation of dopamine D2 autoreceptors, which have been shown to directly modulate dopamine transporter activity. For example, drugs that act upon D2 receptors influence dopamine uptake rates via disruptions in dopamine transporter function, with D2 agonists increasing, and D2 antagonists decreasing dopamine uptake rates (Meiergerd et al., 1993). A similar finding is observed with D2 KO animals, which display decreased dopamine transporter function (Dickinson et al., 1999). These changes in dopamine uptake following D2 manipulations are likely associated with trafficking of dopamine transporters, as it has been shown that the presence of D2 receptors facilitates intracellular dopamine transporters to move to the cell surface (Lee et al., 2007). In this manner it is possible that, by regulating dopamine neuronal firing, hypocretins impact D2 autoreceptor receptor activity, not only affecting the activity state of dopamine neurons, but also D2 related dopamine transporter function and related changes in dopamine uptake.

In either case, it is likely that under baseline conditions, normal levels of hypocretin tone serves to facilitate dopamine neuronal responsivity to afferent signals (e.g., glutamate) such that dopamine neurons will fire and release dopamine at levels appropriate for typical responses to drugs of abuse. Under these circumstances cocaine can exert its typical effects and elevate dopamine levels in target regions. However, when hypocretin neurotransmission is enhanced or compromised, dopamine neuronal activity and release are affected. In the case of reduced hypocretin signaling, (e.g., via SB-334867 treatment), dopamine neuronal activity state would be compromised and thus dopamine release and sensitivity to glutamate would similarly be reduced leading to dysregulation in dopamine transporter function and alterations in the ability for cocaine to exert its effects.

### Summary

The neurochemical studies described above provide compelling evidence for the hypothesis that hypocretin neurotransmission influences dopamine signaling in the NAc core, particularly in response to cocaine. Moreover, given that many of the described experiments used infusions of agents directly into the VTA, these studies indicate that the actions of hypocretin on dopamine release and uptake involve signaling within the VTA and possibly enhancement of glutamatergic signaling within dopamine neurons in this region.

## Hypocretin involvement in reward and reinforcement processes

The mesolimbic dopamine system, including the projection from the VTA to the NAc, is hypothesized to play an integral role in the reinforcing properties of various stimuli including food, sex, and drugs of abuse such as cocaine (Roberts et al., 1977; Woolverton and Johnson, 1992; Robinson and Berridge, 1993; Wise, 1996; Koob and Le Moal, 1997; Volkow and Wise, 2005). Given the observations that hypocretin neurons project to and modulate dopamine function, and that hypocretin manipulations results in altered dopamine responses to drugs of abuse, it is likely that the hypocretin system also participates in the regulation of reward and reinforcement related behaviors. Over the past several years, multiple reports have focused on the extent to which the hypocretin system influences behavioral responses to cocaine and other drugs of abuse, in part, via actions on the mesolimbic dopamine system.

### Hypocretin neurons are activated by drugs of abuse

A number of studies have demonstrated that hypocretin neurons are activated by psychostimulants and other drugs of abuse. For example, acute injections of methamphetamine or nicotine (Ko et al., 2003; Pasumarthi et al., 2006) increase Fos immunoreactivity in hypocretin neurons to three times the levels observed under control conditions. In addition, chronic nicotine also increases hypocretin peptide and hypocretin receptor mRNA levels (Kane et al., 2000). By comparison, cessation of chronic drug delivery and subsequent withdrawal can also elicit activation of hypocretin neurons. Naloxone-induced withdrawal from chronic morphine increases Fos in hypocretin neurons and increases levels of hypocretin peptide mRNA in the NAc shell (Georgescu et al., 2003; Zhou et al., 2006; Sharf et al., 2008). It appears that hypocretin 1 receptors are involved in these actions given that SB-334867 attenuates symptoms of morphine withdrawal and associated changes in Fos in the NAc shell (Sharf et al., 2008). Finally, it has also been shown that hypocretin neurons show increased Fos following presentation of a drug associated cue. In one study using a model of drug relapse, rats that were exposed to an environment previously associated with ethanol reward showed significantly higher Fos levels in hypocretin neurons than animals exposed to a neutral environment (Dayas et al., 2008).

### Conditioned place preference

The previous Fos observations demonstrate that hypocretin neurons are responsive to administration of various drugs of abuse. Whether the increased activity of hypocretin neurons is simply associated with a generalized drug effect on arousal or with more direct actions on reward mechanisms is not fully known. Nevertheless, some studies suggest that general effects of these drugs cannot solely explain the effects of cocaine and morphine on hypocretin neuronal activity. For example, Harris and colleagues demonstrate that hypocretin neurons are preferentially activated in animals that develop CPP for morphine or cocaine and not in those that fail to acquire CPP (Harris et al., 2005; Harris and Aston-Jones, 2006). Specifically, rats that acquired CPP had 3-fold greater numbers of Fos-positive hypocretin neurons than naïve animals or animals that were treated with these drugs but did not acquire CPP. In another set of studies, it was also shown that the degree of Fos activation was proportional to the magnitude of CPP obtained with chemical activation of hypocretin neurons, again suggesting that hypocretin activity is responsive to the rewarding aspects of these drugs.

The importance of hypocretin signaling in reward-related processes is also evident in CPP experiments using SB-334867. For example, systemic SB-334867 has been shown to prevent CPP for amphetamine (Hutcheson et al., 2011) and for morphine (Harris et al., 2005). Furthermore, single infusions of SB-334967 into the VTA block morphine CPP (Narita et al., 2006; Sharf et al., 2010), as do unilateral lesions of the lateral hypothalamus combined with contralateral SB-33467 infusions into the VTA (Harris et al., 2007). Interestingly, while systemic administration of SB-334867 is capable of blocking CPP for morphine (Harris et al., 2005; Sharf et al., 2010) it is unclear as to its effects on cocaine-induced CPP. For example, Sartor and Aston-Jones demonstrate that systemic administration of SB-332867 blocks cocaine-induced CPP in rats (Sartor and Aston-Jones, 2012), while others show a lack of SB-332867 effects on CPP following treatment in mice (Sharf et al., 2010).

A complete disruption of hypocretin signaling also produces deficits in behavioral responsivity to drugs of abuse. Hypocretin KO mice display decreased morphine dependence, reduced locomotor and dopamine responses to morphine, and they fail to exhibit morphine CPP (Georgescu et al., 2003; Narita et al., 2006; Sharf et al., 2010). In preliminary studies we demonstrated that hypocretin KO mice are also less sensitive to the rewarding properties of cocaine (España and Jones, 2010). In those studies, WT mice showed typical levels of CPP for cocaine. In contrast, however, hypocretin KO mice showed a non-significant trend toward conditioned place aversion. When taken together, these CPP results indicate that hypocretin signaling is critical for the regulation of the rewarding properties of cocaine and other drugs of abuse.

### Locomotor sensitization

Locomotor sensitization is characterized by an increase in locomotor responses to a drug challenge after repeated administration of the drug. Although the mechanisms underlying locomotor sensitization remain poorly understood, this paradigm provides a useful metric for investigating the neural bases of long-term behavioral plasticity which are thought to be mediated, in part, by alterations in dopamine systems (Kalivas et al., 1992). Several observations suggest that the hypocretin system is involved in the development of locomotor sensitization for a variety of drugs including cocaine and amphetamine. McPherson and colleagues showed that amphetamine sensitization results in increased activation of hypocretin neurons, an effect that was not observed with acute amphetamine treatment (McPherson et al., 2007). Locomotor sensitization by amphetamine requires activation NMDA receptors in the VTA, which as described above, is influenced by the hypocretin system (Vezina and Queen, 2000; Borgland et al., 2006). Consequently, it is not surprising that locomotor sensitization with amphetamine can be disrupted with SB-334867 injections (Quarta et al., 2010). Similarly, locomotor sensitization to cocaine can be blocked by i.p. or intra-VTA SB-334867 administration when it is delivered prior to exposure to cocaine (Borgland et al., 2006). Like that posited for amphetamine, it is believed that disruption in cocaine locomotor sensitization is associated with the ability of hypocretins to recruit NMDA receptors to the membrane within the VTA (Borgland et al., 2006). In contrast to that seen with cocaine and amphetamine, however, locomotor sensitization to morphine is unaffected by hypocretin 1 receptor blockade, suggesting the likelihood that opiates do not share the same pathway as psychostimulants for the development of locomotor sensitization (Sharf et al., 2010).

### Self-administration

Although multiple behavioral approaches have been used to examine drug-seeking and drug-taking in rodents, i.v. self-administration is frequently considered to be the preferred method for modeling drug abuse. The flexibility of self-administration techniques allows investigators to vary the schedules of reinforcement so as to parse the effects of drug treatments on the acquisition and maintenance phases of drug intake, on the propensity to relapse, and to model specific aspects of cocaine intake, including drug consumption, diurnal variations in cocaine intake, and the motivation to work for cocaine. The following section will discuss recent observations demonstrating the importance of the hypocretin system in self-administration of cocaine and other drugs of abuse.

#### Maintenance

The extent to which hypocretins regulate the reinforcing actions of drugs has been studied across varying schedules of reinforcement. In initial studies, rats were implanted with a jugular catheter and then trained to self-administer cocaine on a fixed ratio 1 (FR1) schedule of reinforcement in which single lever presses resulted in cocaine delivery (Aston-Jones et al., 2009; España et al., 2010). With little restriction to cocaine access, rats are easily able to titrate blood levels of cocaine by spacing injections out over a session, and consequently, responding on an FR1 schedule provides information about an animal's preferred level of cocaine consumption (Norman and Tsibulsky, 2006). After reaching stable levels of cocaine self-administration, rats were treated with vehicle or 30 mg/kg SB-334867 and the number of cocaine injections taken per hour was assessed. Under these conditions, treatment with SB-334867 has no effect on cocaine consumption. A similar lack of effect on FR1 responding was observed when rats were infused with hypocretin-1 directly into the lateral ventricles (Boutrel et al., 2005; España et al., 2011).

**Figure 4:**
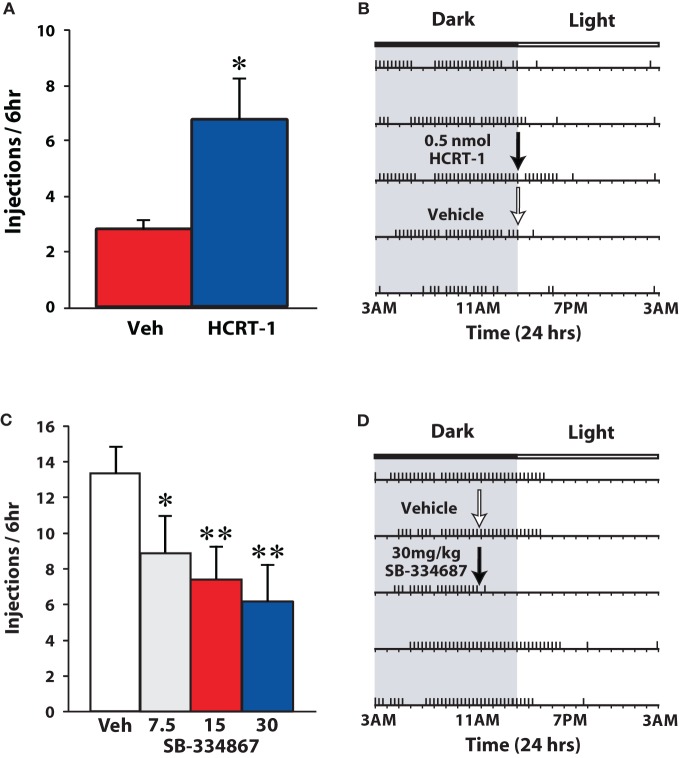
**Hypocretin signaling regulates cocaine self-administration on a DT schedule of reinforcement. (A)** Shown are the mean ± SEM number of cocaine injections taken over the 6 h period following infusion of vehicle (*n* = 7) or 0.5 nmol hypocretin-1 (HCRT-1; *n* = 7). **(B)** Shown is a response pattern from an individual rat that received vehicle (white arrow) or 0.5 nmol hypocretin-1 (black arrow) into the lateral ventricle. **(C)** Shown are the mean ± SEM number of cocaine injections taken over the 6 h period following i.p. injections of vehicle (*n* = 8) or SB-334867 (7.5, 15, and 30 mg/kg; *n* = 8). **(D)** Shown is a response pattern from an individual rat that received an i.p. vehicle (white arrow) or 30 mg/kg SB-334867 injection (black arrow). Horizontal rasters represent 24 h periods. Vertical tick marks represent trials in which a 1.5 mg/kg cocaine injection was taken. Compared to their respective vehicle groups, hypocretin-1 promotes, while SB334867 reduces, cocaine intake. Note that for SB-334867 experiments, rats were treated at 11:00 am when rats are typically awake, whereas for hypocretin-1 experiments, rats were treated at 3:00 pm a time when rats typically discontinue taking cocaine. * *P* < 0.05, ** *P* < 0.01 relative to vehicle. Modified from España et al. (2010, 2011).

These initial observations suggested that active cocaine self-administration was not influenced by the hypocretin system. Nevertheless, several subsequent reports that employed varying schedules of reinforcement demonstrated significant effects of hypocretin manipulations on self-administration of drugs of abuse. For example, blockade of hypocretin 1 receptors decreased responding for nicotine on an FR5 schedule (Hollander et al., 2008; Corrigall, 2009) and ethanol on an FR3 schedule (Lawrence et al., 2006; Richards et al., 2008). The same sensitivity to hypocretin agents is observed when using a 24 h access, discrete trials (DT) schedule in which animals have the opportunity to obtain cocaine only three times per hour. By restricting cocaine access to every 20 min, rats are unable to maximize their blood levels of cocaine and a characteristic pattern of intake emerges in which rats take cocaine almost exclusively during the dark-phase (Roberts et al., 2002; España et al., 2010). Although, the effort requirement is low in a DT paradigm (one lever response results in cocaine delivery), the interaction between dose and availability makes this schedule particularly sensitive to pharmacological and physiological influences which under less restricted access conditions are not observed. As shown in Figure 4, i.c.v. infusions of hypocretin-1 (0.5 nmol) promoted cocaine intake within 20 min of delivery thereby effectively extending cocaine self-administration into the light-phase (España et al., 2011). By comparison, i.p. injections of SB-334867 reduced cocaine intake, particularly in rats treated with the highest dose of SB-334867 (España et al., 2010).

**Figure 5:**
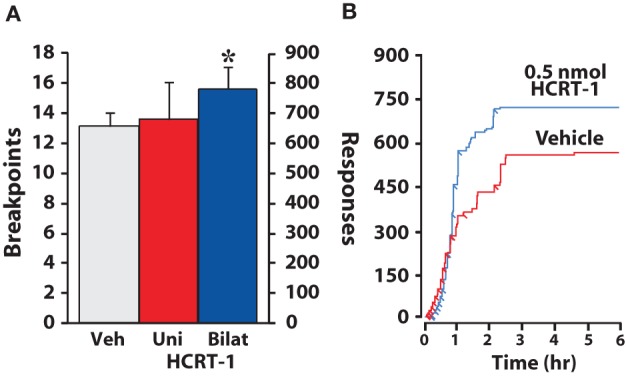
**Hypocretin infusions into the VTA increase the motivation to take cocaine on a PR schedule of reinforcement. (A)** Shown are the mean ± SEM number of cocaine breakpoints and lever responses following unilateral or bilateral intra-VTA infusions of vehicle (uni, *n* = 5; bilat, *n* = 6) or 0.5 nmol hypocretin-1 (HCRT-1; uni, *n* = 5; bilat, *n* = 6). **(B)** Shown are event records from an individual rat that received a bilateral intra-VTA infusion of vehicle or 0.5 nmol hypocretin-1. Cocaine injections are indicated by diagonal tick marks. Relative to vehicle, hypocretin-1 increases the motivation to take cocaine as evidenced by increased breakpoints and lever responding. ^*^ *P* < 0.05 relative to vehicle. Modified from España et al. (2011).

**Figure 6:**
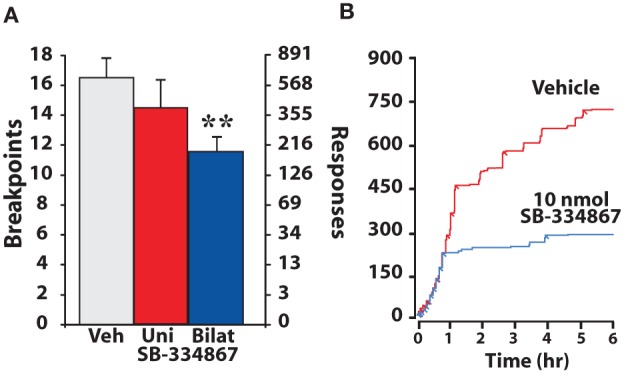
**SB-334867 infusions into the VTA decrease the motivation to take cocaine on a PR schedule of reinforcement. (A)** Shown are the mean ± SEM number of cocaine breakpoints and lever responses following unilateral or bilateral intra-VTA infusions of vehicle (uni, *n* = 5; bilat, *n* = 6) or 10 nmol SB-334867 (uni, *n* = 5; bilat, *n* = 6). **(B)** Shown are event records from an individual rat that received an i.p. injection of vehicle or 30 mg/kg SB-334867. Cocaine injections taken are indicated by diagonal tick marks. Relative to vehicle, SB-334867 decreases the motivation to take cocaine as evidenced by decreased breakpoints and lever responding. ^**^ *P* < 0.01 relative to vehicle. Modified from España et al. (2010).

The progressive ratio (PR) schedule of reinforcement is designed to assess an animal's motivation to work for a reinforcer. In the initial stages of a PR session, single cocaine injections are obtained with relatively low effort (few lever responses) and thus like an FR1 schedule, animals can readily attain preferred blood levels of cocaine. However, as the PR session continues, lever response requirements increase for subsequent cocaine injections and thus rats must exert greater effort to obtain cocaine. The point at which animals discontinue to work for the reinforcer is termed the “breakpoint” and is a measure of the effort that an animal is willing to expend to obtain drug (Richardson and Roberts, 1996). Using the PR schedule, we recently showed that bilateral, but not unilateral intra-VTA infusions of the hypocretin-1 increased the motivation to take cocaine (España et al., 2011). As shown in Figure 5, animals treated with bilateral hypocretin-1 engaged in significantly more lever pressing and reached higher breakpoints than animals treated with vehicle (Figure 5). Consistent with these findings, disruption of hypocretin signaling produces the opposite effect. Thus, both i.p. and bilateral intra-VTA injections of SB-334867 decreased breakpoints and lever response number (Figure 6). These effects are in line with recent reports showing that i.p. SB-334867 reduces PR responding for cocaine, nicotine, or heroin (Hollander et al., 2008; Borgland et al., 2009; Smith and Aston-Jones, 2012). The effects of SB-332867 on PR responding do not generalize to all reinforcers. Although this antagonist reduces breakpoints for cocaine and high-fat food pellets, it has little effect on breakpoints for a regular food reinforcer (Borgland et al., 2009). This is particularly relevant when considering the possible use of hypocretin drugs for the treatment of addiction, as this data suggests that a propensity for an individual to work for drug could be reduced without interfering with the motivation to obtain natural and possibly less salient reinforcer, such as food.

**Figure 7:**
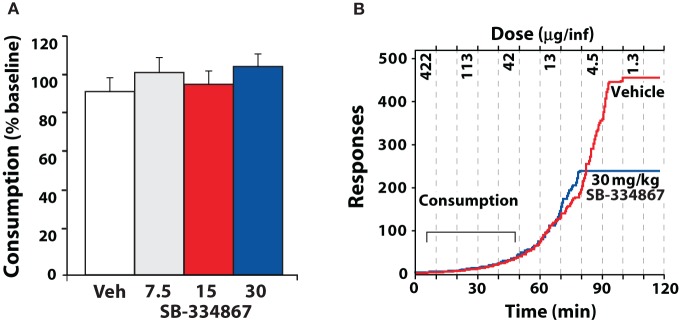
**SB-334867 reduces responding as the unit price of cocaine is increased. (A)** Shown are the mean ± SEM percent baseline consumption of cocaine following i.p. injection of vehicle (*n* = 9) or SB-334867 (7.5, 15, or 30 mg/kg *n* = 9) on the threshold schedule of reinforcement. **(B)** Shown are the event records from an individual rat that received an i.p. injection of vehicle or 30 mg/kg SB-334867. Dashed lines denote times in which cocaine doses were reduced every 10 min (for clarity only every other dose is shown). Note that the rate of responding increases as the dose of cocaine is lowered and eventually rats cease responding for cocaine. During the early portions of the session, SB-334867 has no effect on consumption, however, SB-334867 reduced the dose at which rats ceased responding for cocaine. Modified from España et al. (2010).

The threshold schedule of reinforcement has been used to examine consumption and motivation within a single session (España et al., 2010). During the early portions of a threshold session, single lever responses result in delivery of high doses of cocaine and thus rats can easily attain preferred levels of cocaine. Under these conditions, SB-334867 has no effect on cocaine intake, similar to what is observed with an FR1 schedule. Nevertheless, as the dose of cocaine is lowered across the session, rats must elicit increasingly higher numbers of lever responses to maintain blood levels of cocaine. At this higher price of cocaine, injections of SB-334867 reduce responding earlier in the session, and thus less cocaine intake is observed (Figure 7).

#### Reinstatement

Reinstatement of drug seeking behavior is a useful method to assess the propensity to relapse by measuring a return of operant drug seeking following a period of forced abstinence. Drug seeking can be reinstated by various methods including presentation of drug or context cues, stress, or administration of various pharmacological agents (Crombag et al., 2002; Boutrel et al., 2005; Smith et al., 2010). Several research groups have examined the effects of hypocretin agents on reinstatement of drug seeking behavior. For instance, i.c.v. infusions of hypocretin-1 are sufficient to reinstate cocaine and nicotine seeking (Boutrel et al., 2005; Plaza-Zabala et al., 2010), while peripheral treatment with SB-334967 reduces cue-induced reinstatement of ethanol and cocaine seeking (Lawrence et al., 2006; Smith et al., 2009). This reduction in cocaine seeking could be due, in part, to hypocretin actions in the VTA, as intra-VTA infusions of SB-332867 result in a reduction in cue-induced reinstatement of cocaine (James et al., 2011; Mahler et al., 2012). Further, Mahler and colleagues demonstrate that these effects are due to the ability of hypocretin to facilitate AMPA responses to glutamate inputs related to the cues associated with prior self-administration (Mahler et al., 2012). Although they demonstrate that this is a potential mechanism for cue-induced reinstatement, the same drugs failed to affect cocaine-primed reinstatement, indicating that this is a mechanism specific to the drug paired cues. Furthermore, SB-334867 also attenuates stress-induced reinstatement of cocaine-seeking. In those studies, peripheral SB-334867 reduced reinstatement of cocaine seeking following both foot shock and yohimbine-induced stress (Boutrel et al., 2005; Richards et al., 2008).

### Summary

The self-administration studies described above have shown a series of consistent findings indicating a prominent role for the hypocretin system in the regulation of reinforcement processes. In general, manipulations that reduce hypocretin signaling result in reduced motivation to work for drugs, specifically under conditions where drug availability is limited or where a substantial amount of effort must be expended for an animal to obtain drug. In contrast, under conditions that do not require much effort, and animals can maximize blood levels of drug, hypocretin disruptions do not affect drug intake. The opposite findings are observed when hypocretin signaling is enhanced. This only holds true for highly salient reinforcers, as SB-334867 infusions do not affect the effort that an animal is willing to exert to obtain normal food reward. Thus, infusions of hypocretin-1 increase the motivation to work for drugs under conditions that require effortful responding. Together these observations indicate that the hypocretin system regulates behavioral responses associated with cocaine and other highly salient reinforcers.

## Hypocretin involvement in arousal

**Figure 8:**
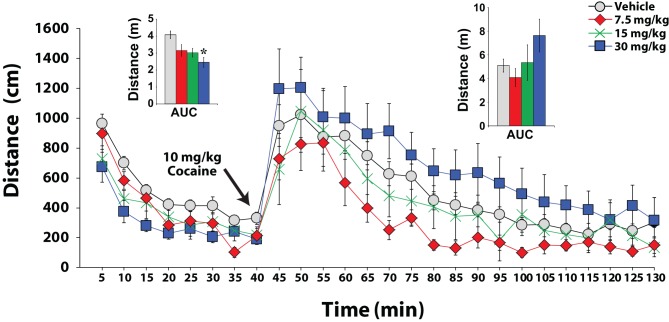
**SB-334867 does not reduce cocaine-induced locomotor activity.** Shown are the mean ± SEM of locomotor activity expressed as distance traveled (cm) for animals treated with i.p. vehicle (*n* = 26) or SB-334867 (7.5, 15, or 30 mg/kg *n* = 9). Left inset shows area under the curve (AUC) during the habituation period. Right inset shows AUC after i.p. injection of 10 mg/kg cocaine. During habituation, SB-334867 reduced locomotor activity but only for the highest dose tested (30 mg/kg). Following i.p. cocaine, however, SB-334867 had no significant effect on locomotor activity. Note that following cocaine injections, the 30 mg/kg SB-334867 group showed the highest amount of locomotor activity. ^*^P < 0.05, relative to vehicle.

Many observations indicate that the hypocretin system regulates arousal-related processes, including locomotor activity and sleep-wake behavior (Hagan et al., 1999; Bourgin et al., 2000; Piper et al., 2000; España et al., 2001). Consequently, it has been posited that hypocretin manipulations influence self-administration via gross deficits in arousal or motor function rather than through actions on reinforcement mechanisms. Despite this possibility, several observations argue against this hypothesis. First, previous studies indicate that while treatment with hypocretin peptides promotes a high arousal state, blockade of hypocretin 1 receptors does not elicit sleep and does not alter responding for natural rewards including water and food (Lawrence et al., 2006; Deng et al., 2007; Rasmussen et al., 2007; Hollander et al., 2008; Borgland et al., 2009; Dugovic et al., 2009). In particular, our work indicates that, in food restricted rats, sucrose self-administration is unaffected by SB-334867 at doses that significantly reduce cocaine intake and sucrose self-administration in sated animals (España et al., 2010). Furthermore, FR1 studies from multiple research groups demonstrate that blockade of hypocretin-1 receptors does not affect the rate of cocaine responding, indicating that rats maintain normal motoric responses on levers (Aston-Jones et al., 2009; España et al., 2010). Lastly, our recent studies demonstrate that locomotor activity in response to cocaine remains unchanged following SB-334867 administration. In those studies, rats were treated with varying doses of SB-334867 (7.5, 15, and 30 mg/kg) and 30 min later were introduced into a novel, open-field, locomotor chamber. Locomotor activity (distance traveled in cm) was measured using automated computer-based calculation of infrared photobeam crossings. After 40 min of baseline locomotor testing, rats were treated with a single i.p. injection of 10 mg/kg cocaine, and locomotor activity was measured for another 90 min. Data were analyzed as area under the curve collapsed across time as independent One-Way ANOVAs for the pre-cocaine acclimation period and for the post-cocaine testing period using SPSS 20 (International Business Machines Corp, Armonk, NY). As shown in Figure 8, during the first 40 min of testing a modest decrease in baseline locomotor activity was observed following SB-334867 treatment [*F*_(3, 41)_ = 6.82, *p* < 0.001]. Nevertheless, a Tukey's *post-hoc* test indicated that this effect was only significant at 30 mg/kg SB-334867 (*p* < 0.001). More importantly, however, when rats were challenged with an i.p. dose of 10 mg/kg cocaine, there were no significant differences in locomotor activity across doses of SB-334867 [*F*_(3, 41)_ = 2.54, *p* < 0.07]. In fact, rats treated with 30 mg/kg showed the highest levels of activity when compared to all other groups, suggesting that when cocaine is on board, rats treated with SB-334867 do not display overall disruptions to arousal or deficits in locomotor activity.

These observations indicate that while hypocretin manipulations may affect some aspects of arousal, they do not produce sedation or locomotor deficits that would explain reductions in behavioral responses to drugs of abuse. Indeed, blockade of hypocretin 1 receptors does not reduce locomotor responses to cocaine, has no effect on lever responding under an FR1 or sucrose-reinforced PR schedule in highly-motivated rats. When considered together, these data suggest that the pharmacological effects of SB-334867 on motivated responding for cocaine cannot be solely attributable to sedation or disruptions to motor activity.

## Conclusions

In less than a decade, nearly a hundred articles have focused on the involvement of the hypocretin system in regulating natural and drug reward. Studies using a variety of electrophysiological, neurochemical, molecular, and behavioral approaches have shown that the hypocretin system is important for normal neurotransmitter and behavioral responses to drugs of abuse. At a gross level, it is evident that enhancement of hypocretin signaling enhances behavioral responses to the rewarding properties of drugs, promotes the motivation for animals to work for drugs of abuse, and increases the propensity for drug seeking and drug taking. By comparison, disruptions in hypocretin signaling reduce drug reward, decrease the motivation to work for drugs, and reduce drug intake and drug seeking.

The effects of hypocretin on behavioral responses to drugs of abuse are likely to be associated with alterations in dopamine neuronal activity in the VTA. Many observations indicate that under normal conditions hypocretins influence dopamine cell firing and dopamine release across regions in the brain that are known to participate in reward and reinforcement processes. Furthermore, it is clear that manipulations to hypocretin neurotransmission also affect dopamine responses to drugs of abuse with enhancement of dopamine release observed with increased hypocretin signaling and reduced dopamine release seen when hypocretin neurotransmission is disrupted. Whether hypocretin regulation of dopamine signaling is a principal participant in the alterations observed with behavioral responses to drugs of abuse is unclear. Nevertheless, given the importance of dopamine systems in regulating reward and reinforcement-related behaviors, it appears that the hypocretin system may be a viable target for pharmacotherapy development to treat drug dependence and relapse without the abuse potential, or intolerability, associated with many of the current treatments for drug addiction. Furthermore, given the evidence that hypocretins also modulate the motivation to work for palatable foods when rats are hungry, but not when they are sated, suggests that the hypocretins may also serve as targets for the treatment of addiction to natural reinforcers, in general, and not only for addiction to drugs of abuse.

### Conflict of interest statement

The authors declare that the research was conducted in the absence of any commercial or financial relationships that could be construed as a potential conflict of interest.
